# Pancreatic Fistula Following Pancreaticoduodenectomy: Clinical Predictors and Patient Outcomes

**DOI:** 10.1155/2009/404520

**Published:** 2009-05-18

**Authors:** C. Max Schmidt, Jennifer Choi, Emilie S. Powell, Constantin T. Yiannoutsos, Nicholas J. Zyromski, Attila Nakeeb, Henry A. Pitt, Eric A. Wiebke, James A. Madura, Keith D. Lillemoe

**Affiliations:** ^1^Department of Surgery, School of Medicine, Indiana University, Indianapolis, IN 46202, USA; ^2^IUSimon Cancer Center, 535 Barnhill Drive, Indianapolis, IN 46202, USA; ^3^Departments of Surgery and Biochemistry and Molecular Biology, Cancer Research Institute, 980 W. Walnut Street R3-C522, Indianapolis, IN 46202, USA; ^4^Department of Biostatistics, School of Medicine, Indiana University, Indianapolis, IN 46202, USA

## Abstract

Pancreatic fistula continues to be a common complication following PD. This study seeks to identify clinical factors which may predict pancreatic fistula (PF) and evaluate the effect of PF on outcomes following pancreaticoduodenectomy (PD). We performed a retrospective analysis of a clinical database at an academic tertiary care hospital with a high volume of pancreatic surgery. Five hundred ten consecutive patients underwent PD, and PF occurred in 46 patients (9%). Perioperative mortality of patients with PF was 0%. Forty-five of 46 PF (98%) closed without reoperation with a mean time to closure of 34 days. Patients who developed PF showed a higher incidence of wound infection, intra-abdominal abscess, need for reoperation, and hospital length of stay. Multivariate analysis demonstrated an invaginated pancreatic anastomosis and closed suction intraperitoneal drainage were associated with PF whereas a diagnosis of chronic pancreatitis and endoscopic stenting conferred protection. Development of PF following PD in this series was predicted by gender, preoperative stenting, pancreatic anastomotic technique, and pancreas pathology. Outcomes in patients with PF are remarkable for a higher rate of septic complications, longer hospital stays, but in this study, no increased mortality.

## 1. Introduction

Despite major advances in pancreatic surgery, pancreatic fistula (PF) continues to be a common complication after pancreaticoduodenectomy (PD) occurring in 9–14% of patients even at high volume centers [1–10]. Classical risk factors associated with PF have been described in the literature and include the following: patient demographics, pancreatic gland texture, pancreatic duct size, pathology of periampullary lesion, anastomotic technique, and surgeon volume [11–20]. This complication with PD continues to occur despite an acknowledged improvement in other types of postoperative complications and declining mortality rates in most large surgical series. As most surgeons realize, the technical challenges of sewing the pancreas to the intestinal tract are formidable. Despite these difficulties, the overwhelming majority of pancreatic anastomoses (80–90%) heals without significant leakage. If substantial progress in reducing the rate of PF is to be made, improved understanding of the risk factors which predispose to fistula formation seems essential. Identifying which glands in which patients are at high risk for a leak can trigger modifications in surgical technique, drainage, and postoperative care to minimize fistula formation. In this study we asked the following questions: first, what preoperative and perioperative factors might predict PF following pancreaticoduodenectomy? Second, what is the effect of PF on outcomes following pancreaticoduodenectomy? To answer these questions, we analyzed our experience with PD at a high-volume pancreatic referral center and then examined the effect of these variables on the incidence of PF and the clinical outcomes in this group of patients.

## 2. Materials and Methods

This is a retrospective review of a case series of consecutive patients who underwent pancreaticoduodenectomy for a broad range of periampullary and pancreatic pathologies between January 1980 and June 2002 at Indiana University Medical Center, a tertiary care academic training hospital in Indianapolis, Indiana. Only patients who had pancreaticoduodenectomy with a remnant, viable pancreatic tail were included for analysis. Patients who underwent total pancreatectomy due to positive resection margins or those who had undergone previous distal pancreatic resections prior to their pancreaticoduodenectomy were excluded from this study. Pancreatic fistula (PF) was defined during the broad range of years in this experience by several definitions. From 1980 to 1985, a PF was defined as >10 mL of amylase-rich fluid per day from a surgically placed drain or through a cutaneous site (around a penrose drain) after postoperative day 8 and lasting for at least 8 days. From 1985–2002 our definition was >50 mL/day of amylase rich fluid after postoperative day 11 and lasting for at least 11 days. Since 2005 at our institution, we have used the International Study Group for Pancreatic Fistula (ISGPF) guidelines defining pancreatic fistula as any measurable volume of fluid on or after postoperative day 3 with amylase content greater than 3 times the serum amylase activity (21). Based on a careful review of the patients medical records during their hospital treatment and postoperative clinical follow-up, pancreatic fistula grades A, B, or C were assigned to each fistula identified as defined in the ISGPF guidelines (see Table 1). Data were collected and reported in strict compliance with patient confidentiality guidelines put forth by the Indiana University-Purdue University Indianapolis institutional review board.

### 2.1. Data Collection

All patients had a baseline history and physical examination recorded. For preoperative variables twelve patient symptoms, physical signs, and associated conditions were included in the database including the following: abdominal pain, back pain, nausea/vomiting, diarrhea, abdominal tenderness, jaundice, diabetes, pancreatitis, abdominal mass, organomegaly, hemepositive stools, and weight loss. The diagnosis of jaundice was based on the presence of scleral icterus on physical examination. The presence of new onset diabetes mellitus was defined as the existence of glucose intolerance necessitating diet modification, oral hyperglycemic drug, or insulin supplementation within two years of the diagnosis of a periampullary pathologic disorder requiring operation. The diagnosis of diarrhea was made if patients reported three or more bowel movements per day or if the total volume of their movements in a day was 0.5 liters or greater. Weight loss was quantified by patient history at the time of the preoperative visit. 

Nine preoperative serum chemistries (total bilirubin, alkaline phosphatase, SGOT, total protein, albumin, amylase, glucose, calcium and phosphorous), five hematologic parameters (hematocrit, hemoglobin, white blood cell count, prothrombin time, and partial thromboplastin time), and three serum tumor marker values (CEA, CA-19-9, and CA-125) were obtained from routine blood draws either in the preoperative clinic or during a hospitalization close to the time of operation. 

Eleven perioperative variables including pre-operative biliary stenting (endoscopic or percutaneous transhepatic), pre-operative transfusion, pre-operative total parenteral nutrition (TPN), pre-operative bowel preparation, estimated intra-operative blood loss (mL), intra- and post-operative transfusions, operative time (minutes), type of resection (standard versus pylorus preservation), type of pancreaticojejunostomy (invagination or end-to-side duct to mucosa), type of intraperitoneal drains used (penrose or closed suction Jackson-Pratt drains), and gastrostomy tube placement were identified from the patient medical records, anesthesiologist's records, and the operative reports.

All specimens were reviewed by the staff pathologist at Indiana University Medical Center with the majority of specimens also undergoing a secondary review by a dedicated pancreatic pathologist. Histopathologic diagnosis, tumor size, tumor differentiation (well, moderate, poor, none), lymph node status (positive, negative), and surgical margin status (positive, negative) were routinely reported. Margins analyzed included the common bile duct, pancreatic neck, and duodenum or stomach. Retroperitoneal (Uncinate) soft tissue margins were inconsistently analyzed early in this series (1980–2002), but since 2002, at our facility, standardized retroperitoneal margin assessment and analysis have been carried out based on AJCC guidelines (37). Clinical outcomes variables that were assessed included the following: in-hospital mortality, overall complications, and specific complications such as cardiopulmonary, delayed gastric emptying (DGE), sepsis, wound infection, intra-abdominal abscess, hemorrhage, and hospital length of stay (LOS). Delayed gastric emptying was defined in this cohort as the inability to take a regular diet after postoperative day no. 10. Survival was determined from the most recent encounter at Indiana University Hospital and was cross-referenced with the Clarian Cancer Registry Database and the Social Security Database. 

### 2.2. Operative Techniques

Twelve surgeons operated on patients included in this study who underwent either pylorus preserving or classic (hemigastrectomy) pancreaticoduodenectomy. Truncal vagotomy was performed in most patients undergoing hemigastrectomy. Reconstruction was undertaken with an isoperistaltic limb of jejunum in retrocolic fashion and anastomosed with an end-to-side pancreaticojejunostomy, followed by an end-to-side choledochojejunostomy and an (antecolic or retrocolic) end-to-side duodenojejunostomy or gastrojejunostomy. The pancreaticojejunostomy was performed using a duct-to-mucosa anastomosis (*n* = 453) or, alternatively, an invaginated anastomosis (*n* = 52) based on surgeon preference. Of the 52 invaginated anastomoses in this series, 6 surgeons used this technique. Only one surgeon used this technique predominately, accounting for 63% of their total PD. During this period at our institution, thirty-five anatomoses (7%) were constructed using pancreatic duct stents. There were no pancreaticogastrostomies used. The pancreaticojejunostomy and choledochojejunostomy were drained routinely with Penrose drains to gravity or closed suction drains. Drainage around Penrose drains was collected with an ostomy skin appliance. Prophylactic octreotide was not routinely used. Finally, some patients in this series underwent elective gastrostomy for postoperative decompression and alimentation.

### 2.3. Statistical Analysis

Statistical associations between categorical factors were assessed using the Fisher's Exact Test. The association of categorical factors with survival was assessed using the Kaplan-Meier Method (KM) and was tested using the log-rank test. The association of continuous variables with survival was analyzed using a Cox's proportional hazards regression model and tested via the Wald Test. Median values of continuous data were compared using the Kruskal-Wallis Test. Analyses were performed with a forward stepwise logistic regression model selection procedure where the probability of inclusion of a factor in the model was set at 20% and the probability of removal factor from the model was 20%. Statistical significance was set at *P* ≤ .05. 

## 3. Results

Of 510 patients undergoing pancreaticoduodenectomy, 46 patients (9%) had a PF. The mean age was the same in patients with PF (58 years; range 30–79) compared to those without PF (58 years; range 15–93). Two hundred eighty four patients (56%) were male. Sixty-five patients underwent pancreaticoduodenectomy in the 1980s with the remainder being performed from 1990 and 2000. The incidence of PF as a function of time by decade (1980's = 9%; 1990's = 6%; 2000's = 10%) or by year (*P* = NS) did not change significantly over the course of the study period. Pancreatic fistula was more common in males, with this difference approaching statistical significance in univariate analysis, (*P* = .06). 

Of the twelve preoperative patient symptoms tracked, none were associated with a significant difference in the rate of fistula formation (data not shown). Notably, a preoperative diagnosis of diabetes was present in a greater proportion of patients who did not have PF; however, this result was not statistically significant. Only serum glucose showed a significant association (*P* = .04) with PF formation. Serum glucose tended to be higher in patients who did not have a PF corroborating the trend in diabetes incidence previously noted.

Eleven perioperative variables that were evaluated are shown in Table 2. Preoperative biliary stenting was done in 211 patients, 68 of whom had percutaneous transhepatic biliary stenting, and 143 had endoscopic stenting. Preoperative biliary stenting (combined percutaneous and endoscopic groups) showed no significant difference in PF rates when compared to patients who were not preoperatively stented (9% stented group (20/211) versus 8% without stenting (26/299)). When subclassified into percutaneous transhepatic or endoscopic stenting however, a significantly higher incidence of PF was found in the percutaneous transhepatic stent group, 16% (11/68) compared to endoscopic stent group 6% (9/143) (*P* = .04). Preoperative blood transfusion or preoperative TPN were not associated with PF formation. Information about preoperative mechanical bowel preparation was not tracked in the latter half of our series; however, among patients in whom preoperative mechanical bowel preparation status was known, a significantly higher incidence of PF was identified in patients who did not undergo mechanical bowel preparation (19% (6/31) versus 6% (14/226), *P* < .02) implying that mechanical bowel preparation may be protective against PF. Average and median blood loss and transfusion requirement were not significantly associated with PF. Median operative time, however, was comparatively longer in the PF group (7 hours versus 5 hours, *P* < .001). Pylorus preserving pancreaticoduodenectomy was performed in 259 patients (51%) and classic pancreaticoduodenectomy was performed in 251 patients (49%). The incidence of PF was not significantly different according to operation performed (11%, (28/259) PPPD versus 7% (18/251) classic). In general, two types of pancreaticojejunostomy reconstruction were performed. A duct-to-mucosa anastomosis was performed in 453 patients, and an invaginated anastomosis was performed in 52 patients. In the remaining 5 patients, 3 underwent Peustow reconstruction of the remnant, and in 2, the reconstruction was unclear based upon operative records. There was a more than 3X higher incidence of PF in invaginated anastomoses (23%) when compared to duct-to mucosa (7%), *P* < .001. Two types of drainage were employed over the course of these operations, closed suction drainage to a bulb suction device usually of the Jackson-Pratt type, and passive/gravity drainage with a Penrose drain. Closed suction drainage was used in 269 patients (53%) and passive drainage with a Penrose drain was used in 241 patients (47%) with both techniques used continuously throughout this study. There was a significantly higher incidence of PF in patients who underwent closed suction drainage (14%) compared to passive Penrose drainage (3%), *P* < .001. Placement of a gastrostomy tube was done in 74 patients and was not associated with a comparatively different PF rate.

Incidence of PF was evaluated according to underlying pancreas pathology (Table 3). The development of a PF was much more common in patients with the diagnosis of duodenal (15%) or Ampullary adenocarcinoma (17%) (*P* = .01 and .06, resp.). Alternatively, patients with a diagnosis of pancreatitis were less likely to develop a PF (*P* = .02). Characteristics of the tumor pathology in patients with periampullary adenocarcinoma are provided in Table 4. Size of pancreatic lesion measured in longest diameter, tumor differentiation, surgical margin status, and lymph node status were assessed. There was no statistically significant difference in any of these variables and the incidence of PF formation. 

Clinical outcomes are given in Table 5. There was a 4% mortality rate in patients who did not have PF and no mortality in patients who had a PF, a difference that was not statistically significant. There were no differences in rate of complications between patients who had a PF when compared to those who did not; however, the total number of complications (number of complications per patient) was greater in patients who developed a PF. In addition, the type of complications varied according to whether or not a PF was present. The incidence of sepsis (*P* < .001), wound infection (*P* < .001), and intra-abdominal abscess (*P* < .001) were significantly higher in the PF group. The 26% reoperation rate for patients with PF was significantly higher than the 0.4% rate in patients without PF. Of the twelve reoperations done in the PF group, eleven were done for complications of the PF and only one was done for a persistent fistula which lasted for greater than 4 months. Six reoperations were done prior to the diagnosis of PF for sepsis or a visceral pseudoaneurysm, and six were done in patients following the diagnosis of PF for complications including the following: wound dehiscence (1), poor fistula drainage with sepsis (3), breakdown of the hepaticojejunostomy (1), and persistent fistula >4 months (1). The diagnosis of PF was made on average on postoperative day 8 and these patients experienced longer lengths of hospital stay. The median length of stay in the PF group was 25 days, whereas it was only 12 days in the no PF group (*P* < .001). Based upon international study group for pancreatic fistula (ISGPF) grading guidelines, this series included 10 grade A fistulas, 23 grade B fistulas, and 13 grade C fistulas [21]. Forty-five of 46 PF (98%) closed without reoperation with a mean time to closure of 34 days (range 3–93 days). Twenty-three patients (all grade 13 grade C fistulas and 10 grade B fistulas) were treated with antibiotics. Ten patients (5 grade C and 5 grade B fistulas) were treated with octreotide. This timing of closure was not significantly different with/without octreotide treatment (39 ± 5 versus 25 ± 5) or with/without antibiotic treatment (36 ± 5 versus 29 ± 6). Thirty-six patients required TPN for an average of 32 days (range 5–90 days). Time to closure was not affected by nutrition route (enteral versus parenteral), daily PF output, or the level of amylase/lipase in the PF fluid analysis. Average daily fistula output was 163 ± 26 cc but with a wide range (50–2300 cc). Computed tomography (CT) directed percutaneous drainage of an intra-abdominal fluid collection was required in 11 of 46 patients with a PF. In seven of these patients, the fistula grew bacteria on routine culture. Notably, there was a trend of delayed fistula closure when patients required percutaneous drainage of any intra-abdominal fluid collection postoperatively (42 ± 7 days with versus 30 ± 5 days without, *P* = .09).

A multivariate analysis was performed of all parameters with a forward stepwise logistic regression model (Tables 6(a)–6(c)). This analysis indicated that independent predictive factors for the formation of a PF were as follows: invaginated anastomosis (OR 3.30, *P* = .01) and closed suction drainage (OR 2.24, *P* = .05). A diagnosis of pancreatitis (OR 0.22, *P* = .05) and preoperative endoscopic biliary stenting (OR 0.34, *P* = .05) were independent protective factors against PF. Because the type of drain was highly correlated with individual surgeon, in a separate assessment we performed a multivariate analysis excluding drain type (Table 6(b)). In this analysis, invaginated pancreaticojejunostomy (OR 4.56, *P* = .002) remained a significant predictive factor of PF and the diagnosis of pancreatitis (OR 0.22, *P* = .05) remained an independent protective factor. While the presence of an endoscopic stent did not reach significance, the presence of a preoperative percutaneous transhepatic biliary stent (OR 2.43, *P* = .07) approached significance. 

A multivariate analysis was also performed on the subgroup of patients with periampullary adenocarcinomas (Table 6(c)). The only independent predictor of PF formation in this group was an invaginated pancreaticojejunostomy (OR 11.8, *P* = .0002). Female gender (OR 0.238, *P* = .03) and endoscopic stent (OR 0.194, *P* = .04) were independent protective factors against PF formation. Diabetes (OR 0.146, *P* = .07) demonstrated a trend as a protective factor against PF formation.

## 4. Discussion

In this study, we report our experience with PF in a series of 510 consecutive pancreaticoduodenectomies performed over a 22-year period. Our incidence of PF was 9% which appears to be comparable to the PF rate of 9–14% reported in other specialized centers [1–10]. It is difficult to compare the incidence and severity of PF without standardization of the diagnosis, terms, and severity of this complication. It is hopeful that the standardized definitions of PF as put forth by the ISGPF will likely allow for more equitable comparison among institutions [21]. 

The risk of PF formation appears to be multifactorial involving demographic, preoperative, intraoperative, and pathologic factors. One prior study has reported that male sex was a significant predictor of PF [22]. In this study, male gender only approached significance as a univariate predictor of PF formation in the overall group of patients undergoing pancreaticoduodenectomy. In our periampullary adenocarcinoma subgroup analysis, females had a lower incidence of PF and these results showed a trend as an independent predictor of PF on multivariate analysis. Advanced age cited by some as predictive of PF was not recognized as a predictor in this series [23, 24].

A higher incidence of PF was identified in patients who did not undergo mechanical bowel preparation. This association to our knowledge has not been reported before and so is an intriguing finding. Perhaps the absence of intestinal succus or stool in the colon minimizes pressure in the jejunum limb translating into reduced PF rates. This association was not significant on multivariate analysis. Intraoperative blood loss cited by some as predictive was also not recognized as a significant factor in this series [23, 24]. Type of pancreaticojejunostomy anastomosis has also been cited by others in the literature to be a predictor of PF [25]. This study showed that the type of pancreaticojejunostomy reconstruction (invaginated versus end-to-side) was significantly associated with PF in both the whole group and periampullary adenocarcinoma subanalysis. A recent metaanalysis addressing this particular association found a higher incidence of PF in patients who had invaginated pancreaticojejunostomy (26%) versus a duct-to-mucosa (16%) type of anastomosis [25]. These findings are in contrast to a randomized control trial of 144 patients performed in Italy by Bassi et al. [26] which showed no significant difference between invaginated and duct-to-mucosa anastomoses in PD. Our pancreaticobiliary group is currently participating in a prospective randomized trial with Thomas Jefferson University to determine whether or not there is a difference in PF rate between invaginated and duct-to-mucosa pancreaticojejunostomy in PD. The results of this trial should be forthcoming later this year (2009).

Preoperative biliary stenting has been an area where some groups show an association with PF. A study from Hopkins suggests that preoperative stenting is associated with an increased risk of PF formation [27]. Our data is variable according to whether the preoperative stenting was endoscopic or percutaneous. On the one hand, preoperative endoscopic stents are actually protective in our subgroup of patients with periampullary adenocarcinoma, while those patients who had preoperative percutaneous transhepatic biliary (PTB) stenting showed an increased risk of PF formation. Other authors have also shown that preoperative endoscopic stenting decreases the incidence of postoperative PF [28].

Our study also suggests that closed suction drainage may be a predictor of PF formation. The Memorial Sloan Kettering group has shown a lower incidence of PF in patients who did not have intraperitoneal drainage following PD [29]. In our series both Penrose drains and closed suction drains were used and the Penrose drain group showed a lower incidence of PF formation. This finding implies that active suction drainage may have a deleterious effect on PF formation. A confounding factor in our study was that the Penrose type drain was used largely by the highest volume surgeon in our series who also had a comparably low PF rate. Drain type covaried so tightly with individual surgeons such that it was not possible to separate whether drain type or individual surgeon was more predictive in our multivariate model. 

One factor which appears to be a consistent is that the texture of the gland is significantly associated with PF formation. Yeo et al. [19] stratified patients in a trial according to the texture of the gland and found that patients with a firmer texture gland had a lower incidence of PF. Other studies have also supported the association between soft pancreatic gland texture and the risk of PF [25, 30, 31]. Our data is indirectly supportive that the texture of the gland is predictive of PF formation in those patients with both duodenal and ampullary adenocarcinomas, who often have a normal (i.e., soft) gland texture; both had a higher incidence of PF formation in our series but intraoperative assessment of the gland at the time of operation was not available in our retrospective database. Other investigators have associated a small pancreatic duct as a contributing factor to PF formation [22, 32]. Pancreatic duct size was not prospectively tracked in our series of patients although it should be noted that this particular variable is often difficult to separate between soft textures of the gland because typically soft glands also have small pancreatic ducts [19, 22]. 

Although not addressed specifically in our analysis, individual surgeons tend to vary widely in their postoperative rate of PF. In particular, two specific surgeons in this series (out of the 12 participants) had significantly different PF rates from the average of their remaining peers. One surgeon had a significantly lower incidence of PF (4% compared to 14% for all others combined, *P* < .001) while the other surgeon had a significantly higher incidence of PF (18% versus 8% for all others combined, *P* = .04). While these differences were accounted for, in part, by the differences in pancreatic pathology in each surgeon's cohort, this issue brings up the surgeon as a significant variable in the incidence of PF formation. 

Pancreatic fistula remains the “Achilles heel” of pancreaticoduodenectomy and novel approaches continue to be put forward to reduce its incidence [33–36]. Although historically, PF were associated with a mortality rate of 20 to 40% [3, 7, 8], current advances in radiologic imaging and interventional radiologic techniques, antibiotics, and critical care medicine have considerably reduced the mortality rates from PF. Our data is consistent with this trend since in our series of patients with PF, there were no in-hospital mortalities. Nonetheless, PFs continues to cause significant morbidity, prolonged hospital stay, and increased hospital cost. Our data supports these observations since patients with PF had a greater total number of complications, a higher incidence of septic complications, and a higher reoperation rate. In addition, our patient's length of hospital stay was twice that of patients who did not have a PF. 

Based on univariate analyses, gender, diabetes, preoperative glucose level, length of operation, bowel preparation, biliary stenting (endoscopic versus percutaneous), anastomotic technique (invaginated versus duct-to-mucosa), intraperitoneal drain choice (passive/gravity versus closed-suction), and pathology (pancreatitis, duodenal cancer) may influence PF formation. In multivariate analyses, invaginated anastomosis, closed suction drainage, and percutaneous biliary stenting all increased the risk of PF while pancreatitis, endoscopic biliary stenting, and female gender conferred protection against PF. The influence of the individual surgeon on PF is also an extremely important factor to consider although difficult to separate from other variables such as gender, pancreatic pathology, or biliary stenting in a retrospectively designed study. 

In conclusion, in this series the development of PF following pancreaticoduodenectomy was predicted by demographics (gender), preoperative procedures (biliary stenting), intraoperative technique (anastomosis, drainage, individual surgeon), and pancreatic pathology (pancreatitis). Outcomes in patients with PF are remarkable for a higher rate of septic complications, an increased incidence of reoperations, and a longer hospital stay without significant difference in overall mortality rate. Preoperative biliary stenting, type of pancreaticoenteric anastomosis, and intraperitoneal drain usage are controllable factors in PF formation that surgeons should continue to investigate to reduce its incidence following PD.

## Figures and Tables

**Table 1:** Parameters utilized to grade post-operative pancreatic fistulas (POPFs) (21).

Grade	A	B	C
Clinical condition	Well	Often well	Ill appearing/bad
Specific treatment*	No	Yes/No	Yes
US/CT (if obtained)	Negative	Negative/Positive	Positive
Persistent drainage (after 3 weeks)^†^	No	Usually yes	Yes
Reoperation	No	No	Yes
Death related to POPF	No	No	Probably yes
Signs of infection	No	Yes	Yes
Sepsis	No	No	Yes
Readmission	No	Yes/No	Yes

US = Ultrasonography; CT = computed tomography scan. *Partial (peripheral) or total parenteral nutrition, antibiotics, enteral nutrition, somatostatin analogue, and/or minimally invasive drainage. ^†^With or without a drain in situ.

**Table 2:** Relationship of measured perioperative variables with the development of a PF in 510 patients who had pancreaticoduodenectomy.

Variable	PF	No. PF	*P*-value
	(*n* = 46)	(*n* = 464)	
Pre-op stent	43% (20)	41% (191)	*P* = NS
* *Endoscopic	20% (9)	29% (134)	*P* = .22
* *Percutaneous	24% (11)	12% (57)	*P* = .04
Pre-op transfusion	6% (2)	5% (18)	*P* = NS
Pre-op TPN	16% (5)	22% (78)	*P* = NS
Pre-op bowel preparation*	43% (20)	50% (237)	*P* = .02
* *Yes	30% (14)	45% (212)	
* *No	13% (6)	5% (25)	
Blood loss (median)	1300 mL	1300 mL	*P* = NS
Transfusions (median)	1 U	1 U	*P* = NS
Operative time (median)	7 hours	5 hours	*P* < .001
Type of resection			*P* = NS
* *Pylorus preserving	61% (28)	50% (231)	
* *Classic	39% (18)	50% (233)	
Type of anastomosis			*P* < .001
* *Duct-to-mucosa	74% (34)	90% (419)	
* *Invaginated	26% (12)	9% (40)	
* *Other (3)/unknown (2)	0 (0)	1% (5)	
Drain type			*P* = < .001
* *Penrose	17% (8)	50% (233)	
* *Closed suction	83% (38)	50% (231)	
G-tube placement	22% (10)	14% (64)	*P* = NS

PF: Pancreatic fistula; *Pre-op bowel preparation information was available in only 257 patients. ( ): Total number of patients in each group with defined variable.

**Table 3:** Primary pathologic diagnosis in 510 patients having pancreaticoduodenectomy.

Variable	PF	No. PF	*P*-value
	(*n* = 46)	(*n* = 464)	
Periampullary Ca	65% (30)	56% (264)	*P* = NS
* *Pancreatic	28% (13)	40% (188)	*P* = NS
* *Ampullary	17% (8)	8% (39)	*P* = .06
* *Duodenal	15% (7)	5% (24)	*P* = .01
* *Bile duct	4% (2)	3% (13)	*P* = NS
Pancreatitis	9% (4)	23% (106)	*P* = .02
Cystic neoplasms	11% (5)	12% (53)	*P* = NS
* *IPMN	4% (2)	11% (47)	*P* = NS
* *Mucinous cystadenoma	2% (1)	1% (4)	*P* = NS
* *Serous cystadenoma	4% (2)	0.4% (2)	*P* = NS
Islet cell neoplasms	6% (3)	3% (16)	*P* = NS
* *Functional	4% (2)	1% (6)	*P* = NS
* *Nonfunctional	4% (2)	1% (6)	*P* = NS
Trauma	4% (2)	1% (6)	*P* = NS
Other	4% (2)	4% (19)	*P* = NS

**Table 4:** Tumor size and histopathologic characteristics in the 294 patients in this series with periampullary malignancies and their relationship to PF formation.

Variable	PF	No. PF	*P*-value
	(*n* = 30)	(*n* = 264)	
Diameter (mean ± SD)	3.2 ± 1.0 cm	3.1 ± 1.5 cm	*P* = NS
Tumor differentiation			*P* = NS
* *Well	8% (2)	8% (22)	
* *Moderate	48% (14)	39% (102)	
* *Poor	22% (7)	31% (82)	
* *None	22% (7)	22% (58)	
Surgical margin status			*P* = NS
* *Negative	80% (24)	83% (219)	
* *Positive	20% (6)	17% (45)	
Lymph node status			*P* = NS
* *Negative	43% (13)	43% (114)	
* *Positive	57% (17)	57% (150)	

**Table 5:** Morbidity, mortality, and length of hospital stay in 510 patients who had pancreaticoduodenectomy.

Outcomes	PF	No. PF	*P*-value
	(*n* = 46)	(*n* = 470)	
Mortality	0%	4%	*P* = NS
LOS (median)	25 days	12 days	*P* < .001
Complications (# patients)	17 (37)	176 (37)	*P* = NS
* *Cardiopulmonary	8 (17)	69 (15)	*P* = NS
* *DGE	3 (7)	35 (7)	*P* = NS
* *Sepsis	10 (21)	22 (5)	*P* < .001
* *Wound infection	9 (20)	17 (4)	*P* < .001
* *Intra-abdominal abscess	7 (15)	10 (2)	*P* < .001
* *Hemorrhage	3 (7)	15 (3)	*P* = NS
Reoperation*	12 (26)	2 (<1)	*P* < .005
* *Reoperation (prior to diagnosis)	6 (13)	NA	
* *Reoperation (following diagnosis)	6 (13)	NA	

PF: Pancreatic fistula; Mortality: In-hospital mortality; LOS: Hospital length of stay; DGE: Delayed gastric emptying; *Reoperation was broken down into reoperations done prior to the diagnosis of PF and following the diagnosis of PF; only one PF was reoperated on for failure to close within 4 months.

**(a) Multivariate analysis of factors predictive of pancreatic fistula.** 

Predictive factor	Odds ratio	*P*-value
Invaginated pancreaticojejunostomy	3.30	.01
Closed suction drainage	2.24	.05
Diagnosis of pancreatitis	0.22	.05
Pre-op endoscopic biliary stent	0.34	.05

**(b) Multivariate analysis of factors predictive of pancreatic fistula excluding transperitoneal drain type.** 

Predictive factor	Odds ratio	*P*-value
Invaginated pancreaticojejunostomy	4.56	.002
Pre-op PTB stent	2.43	.07
Diagnosis of pancreatitis	0.22	.05

PTB: Percutaneous transhepatic biliary

**(c) Multivariate analysis of factors predicting pancreatic fistula in 294 patients with periampullary adenocarcinoma subgroup.** 

Predictive factor	Odds ratio	Stats
Invaginated pancreaticojejunostomy	11.78	.0002
Pre-op endoscopic biliary stent	0.194	.04
Gender (Female)	0.238	.03
Pre-op diabetes	0.146	.07
